# Stopping Interferon Beta 1b Does Not Influence the Risk of Disability Accrual in Non-Active SPMS: Results from an Italian Real-World Study

**DOI:** 10.3390/ijerph19106069

**Published:** 2022-05-17

**Authors:** Aurora Zanghì, Emanuele D’Amico, Francesco Patti, Carlo Avolio

**Affiliations:** 1 UOC Neurology, Sant’Elia Hospital, 93100 Caltanissetta, Italy; a.zanghi@asp.cl.it; 2 Department of Medical and Surgical Sciences, University of Foggia, 72100 Foggia, Italy; c.avolio@unifg.it; 3 Department “G.F. Ingrassia”, MS Center, University of Catania, 95125 Catania, Italy; patti@unict.it; 4 Multiple Sclerosis Center, Department of Neurosciences, Policlinico Riuniti Hospital, 72100 Foggia, Italy

**Keywords:** secondary progressive multiple sclerosis, disease modifying treatment, interferon beta 1b, EDSS

## Abstract

Background: No consensus exists on the possibility to stop disease modifying therapies (DMTs) in Secondary Progressive Multiple Sclerosis (SPMS). Methods: The primary outcome was the time to reach 24-weeks confirmed Expanded Disability Status Scale (EDSS) 7.0. We enrolled all patients with a confirmed diagnosis of non-active SPMS (here, absence of clinical or radiological activity for at least 24 months before the conversion) between 1 January 2010 and 31 December 2015, at MS centers of Catania and Foggia, Italy. Patients were divided into two groups, according to the shared decision to stop DMTs (group A) or to maintain/switch to licensed interferon beta 1b for SPMS (group B). A Cox model adjusted with an inverse probability weighted propensity score (IPTW-PS) was employed. Results: A cohort of 311 patients was enrolled, 165 were in group A and 146 were in group B. Patients in the two groups were similar for baseline characteristics. The IPTW-PS adjusted Cox model for the event time to 24-weeks confirmed EDSS 7.0 did not show differences between the two groups (ExpB 0.96, CI 0.739–1.271, *p* = 0.819). Conclusions: In a real-world setting, in patients with non-active SPMS, the maintaining or switching to the licensed interferon beta 1b did not reduce the risk of reaching confirmed EDSS 7.0.

## 1. Introduction

In recent years, we have witnessed impressive changes in the therapeutic armamentarium of Multiple Sclerosis (MS), but almost confined to relapsing remitting MS (RRMS) [1].

A better understanding of the pathological mechanisms that drive neurodegeneration in individuals with MS is needed to develop therapies that will effectively treat patients in the primary and secondary progressive (SPMS) stages of the disease [2,3].

In early MS, the inflammatory demyelinating process triggers a cascade of events that lead to neurodegeneration and are amplified by pathogenic mechanisms related to brain ageing and accumulated disease burden [2,4,5].

The therapeutic possibilities for SPMS are still scarce even if great expectations are placed on new drugs [6,7] but evidence in real-world practice is still lacking.

No universal guidelines exist about the ability to maintain or withdraw disease modifying therapies (DMTs) in patients with SPMS, and in a real-world setting, the plan of therapy is shared case by case among the patients and treating neurologists [8].

In RRMS, extensive axonal loss occurs in acute lesions, but the clinical deficits of these inflammatory events are mostly reversible as the human brain has a remarkable ability to compensate for neuronal loss.

With time and ongoing inflammatory activity, axonal loss can drive the conversion of RRMS to SPMS when the brain exhausts its capacity to compensate for further neuronal loss.

However, entering into SP phase represents an insidious change. Indeed, the disability accrual becomes irreversible, and the ability to recover from clinical relapses begins to be frequently ineffective [9].

Then inherent difficulty in diagnosing transition is combined with the difficult meaning for patients and physicians further challenging the communication.

Furthermore, few data are available about the course of long-term disease on patients who maintained or switched to the licensed interferon beta 1b [10].

Some evidence seems to suggest that the maintenance of DMTs in small cohorts of SPMS with residual clinical and radiological disease activity could help in delaying severe disability milestones, but no consensus exists, and no insights have been provided about the role of therapy in patients who did not show clinical and radiological activity, (the so-called ‘non-active’ SP patients) [11,12].

We retrospectively investigated whether the withdrawal or maintenance/switch to licensed nterferon beta 1b could influence the risk of reaching severe disability milestones in a large cohort of patients with non-active SPMS for at least 24 months before their conversion to the progressive course.

## 2. Methods

### 2.1. Participants

In this retrospective study, we collected data from patients at the MS centres of Catania and Foggia, Italy, who received diagnosis of SPMS from 1 January 2010 to 31 December 2015. 

The inclusion criteria were: (1) to be patients in the SP course according to criteria from Lorscheider et al. [13]; (2) having an EDSS score at the time of SP conversion between 4.0 and 6.0; and (3) to have not experienced clinical (relapses) and radiological activity (new/enlarging lesions on T2 or T1 gadolinium weighted sequences) for at least 24 months before their conversion to SPMS (non-active).

Patients were also required to have a minimum of 24 months between MS diagnosis and conversion to SPMS and 12 or more months of prospective follow-up after SPMS conversion.

Patients with insufficient baseline clinical and radiological data or insufficient data quality were excluded.

In our study, each patient who moved to SPMS was offered the possibility, according to Italian medicine agency rules, to be treated with interferon beta 1b, which is licensed for the treatment of SPMS.

The decision to stop DMTs in the cohort of non-active SPMS patients was reached after a consultation between the treating neurologists and the patient about the risks and benefits of such a decision. Psychological support was also offered.

Patients were divided into two groups according to the shared decision to withdraw DMTs (group A) or to be treated with licensed interferon beta 1b (250 µg /mL, every other day subcutaneously) (group B) (maintain/switching to).

### 2.2. Protocol Approval Standard, Registrations, and Patient Consents

The study protocol was approved by the local ethics committee (Comitato Etico Catania 1) of the coordinating centre (Policlinico Vittorio Emanuele, Catania, Italy), and patients provided written informed consent. The study was conducted in accordance with the ethical principles of the Declaration of Helsinki and with the appropriate national regulations.

### 2.3. Data Dollection

A retrospective analysis of longitudinally collected data was performed. 

Demographical, clinical and radiological data were extracted from the electronical medical records on Imed^®^ (Merck Serono, Geneva, Switzerland) on 30 September 2020.

Disability level was assessed by Expanded Disability Status Scale (EDSS) and recorded by trained neurologists with an online Neurostatus certification (https://www.neurostatus.net/, accessed on 1 January 2022).

### 2.4. Outcomes

The primary study outcome was to examine the association between the maintenance/withdrawal of DMTs and the achievement of the 24-weeks confirmed EDSS 7.0. 

EDSS 7.0 is referred to a patient who is unable to walk beyond approximately 5 m even with aid and is essentially restricted to a wheelchair.

During the follow up, we also recorded the presence of disease activity in both cohorts, in terms of clinical relapse and/or new enhancing brain lesions in brain Gadolinium T1-weighted sequences.

A relapse was defined as a neurologic deficit related with an acute inflammatory demyelinating event that lasted at least 24 h in the absence of infection or fever.

We routinely decided to not consider new or enlarging lesions on brain T2 weighted sequences due to the evidence in the literature that lesion load in brain T2 sequences has consistently shown a weak association with clinical measures of disease severity, especially in SPMS [14].

### 2.5. Statistical Analysis

All patient characteristics summary statistics are reported in terms of frequencies (%) for categorical variables, the mean standard deviation (S.D.), or median with an interquartile range (IQR) for continuous variables. A parametric or nonparametric test was employed according to results obtained from the Kolmogorov test.

A survival analysis with univariate Kaplan Meier and multivariate Cox proportional were built for the event time to 24 weeks confirmed EDSS 7.0. 

To consider the imbalance of the 2 groups, a propensity score (PS) was calculated as the following.

A logistic regression was performed to score all patients according to the group (0 = No DMT, 1 = Yes DMT) used as an independent variable and the following covariates at baseline: age at disease progression, EDSS one year before disease progression and at the time of SP diagnosis confirmation, number of therapeutic switches before progression, and follow up duration.

Inverse probability of treatment weight (IPTW) and the stabilized inverse probability of treatment weight (SIPTW) were also calculated.

A generalized Cox regression model IPTW PS-adjusted was built for the event time to 24 weeks confirmed EDSS 7.0. In the multivariable analysis, all the variables with a *p*-value < 0.10 in the univariate analysis were considered. The results were presented as an estimate of the hazards ratio (HR) and the corresponding 95% confidence interval (CI). 

A sensitivity analysis was conducted for patients with at least 36 months of follow up (in group B) and without disease activity during the mentioned follow up.

The model with the best statistical properties was chosen according to Akaike criterion.

Statistical significance was taken to be at the two-tailed 0.05 level. All statistical analysis was performed using SPSS 21.0 (SPSS Inc., Chicago, IL, USA).

## 3. Results

At the date of data extraction, out of 2300 patients registered in our records, 311 SPMS patients fulfilled the required criteria to be enrolled. There were 165 patients in group A and 146 in group B. The baseline data are shown in Table 1. The two groups were similar for baseline characteristics. In group B, 130 patients had at least 36 months on therapy and the overall mean time on therapy was 55.2 ± 26.2 months. All patients in group B were previously treated with either glatiramer acetate or IFNs (32/146 on glatiramer acetate, 38/146 on other IFNs, 76/146 on interferon beta 1b).

**Figure 1:**
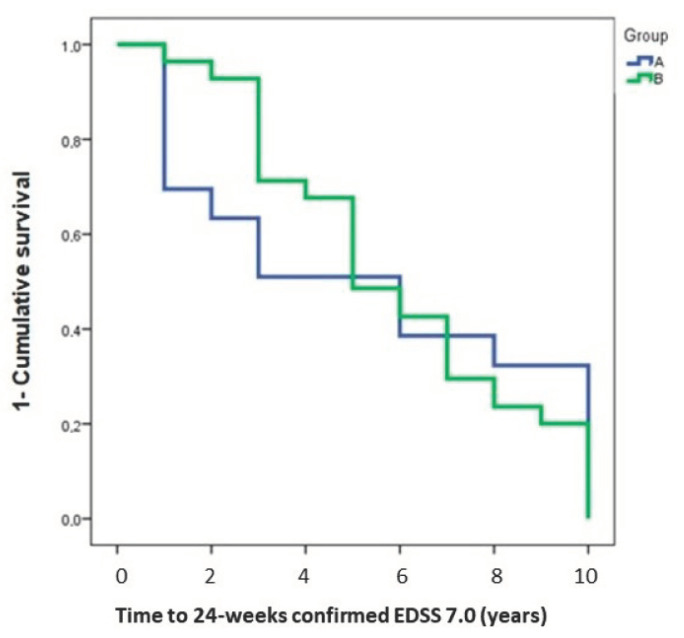
Time to 24-weeks confirmed EDSS 7.0 between the two groups. EDSS: Expanded Disability Status Scale.

The survival model with Kaplan Meier analysis (log rank 0.78, *p*-Value = 0.234) and the Cox proportional model IPTW PS adjusted did not show differences between the two groups for the time to 24 weeks confirmed EDSS 7.0 (ExpB 0.96, CI 0.739–1.271, *p* = 0.819) (Figure 1). A sensitivity analysis for patients with at least 36 months of follow up (127/165 in group A and 135/146 in group B) confirmed the results (ExpB 1.04, CI 0.607–1.798, *p* = 0.874).

During the follow up, six patients (four in group A and two in Group B) had clinical relapses, with a median time of 17.6 ± 7.9 in Group A and 15.8 ± 6.8 months in group B. Seven had radiological activity (three in group A and four in group B 14.5 ± 6.5 and 15.1 ± 5.8 months).

## 4. Discussion

In our real-world study, interferon beta 1b did not influence the risk to reach 24-weeks confirmed EDSS 7.0 in a cohort of patients with non-active SPMS. Furthermore, no differences were observed in acute disease recurrences between the two groups. 

The role of DMT maintenance in SPMS is constantly debated in a real-world setting; however, it is mainly investigated in heterogeneous cohorts. We included only non-active SPMS patients who maintained therapy for a long time (90% for at least 36 months) and all analyses were adjusted for baseline characteristics with IPTW PS.

A recent study from the MSBase study group shows that only patients with superimposed relapses during SPMS may benefit from continued treatment with DMTs, in accordance with the 2018 American Academy of Neurology recommendation. However, no consensus exists, as another report from the same study group has not found any benefits of DMTs use when analyses were adjusted for relapse rate [12,15,16]. This great variability of results raises the need for robust and reproducible studies, even in the observational setting, with more homogeneous cohorts.

DMT efficacy in a cohort of SPMS patients has been investigated in a previous short report that compared a group of patients without evidence of disease activity before SPMS conversion who had withdrawn DMTs (in accordance with a treating neurologist) to another group of patients who stopped DMTs voluntarily [11]. Here, patients with non-active SPMS showed low recurrence (11.7%) of acute disease activity after the discontinuation of DMTs. These results, although in accordance with ours, were not adjusted for baseline characteristics such as age. If they were adjusted, the results could be biased by the different baseline data of the two cohorts [11].

Overall, the clinical dilemma of whether or not to maintain DMTs in such a cohort of SPMS remains unsolved, and neurologists should carefully weight the benefit–risk ratio. 

It is fundamental to emphasize the criticality of the conversion from a RR to a SP disease course, not only because such a conversion is evidence of disability progression, but also because, until recently, treatments that effectively reduced disability progression in relapsing MS were not proven to be effective in SPMS. Early identification of SPMS will require tools that, together with the use of appropriate treatments, may result in better long-term outcomes for the population of patients with SPMS.

We undoubtedly need a better characterization of SPMS cohorts to address our therapeutic efforts. 

There is a need for additional research examining the psychosocial impact of disease progression in order to inform decision-making around policy and care choices.

Furthermore, the formulation of an SPMS diagnosis is a key prognostic factor that may affect decisions and planning at the health care level (e.g., DMT change or discontinuation, the provision of psychosocial support, the shift to multidisciplinary care) [16] and the personal level [17]. Sharing the period of diagnostic uncertainty with the patient, which can take some years, can prepare the patient to receive a confirmed SPMS diagnosis, preventing unexpected and inapt disclosure, as revealed by other studies [18].

A strength of our study is that we have employed a generalized model of IPTW PS-adjusted for baseline characteristics to mitigate unbalance among the groups, and provide a long follow up.

However, our study has some limits. PS cannot balance unselected bias that could be related to the unknown characteristics of each patient, according to disease history and also to demographical/social/economic elements.

As this is a retrospective study, not all participants have the same follow-up, and the characteristics of sample size warrant cautious interpretation of the data.

Our findings seem to suggest that discontinued interferon beta 1b could be in non-active SPMS, but more randomized, prospectively collected data with long-term follow up are needed.

## Figures and Tables

**Table 1:** Demographical and clinical characteristic of the two groups.

Factors *	Group A (165)	Group B (146)	*p*-Value
Male *n* (%)	88 (53.3%)	80 (54.8%)	ns
Female *n* (%)	77 (46.7%)	66 (45.2%)	ns
Age (years)	57.3 ± 9.5	55.8 ± 10.9	ns
Level of education			
Primary level (≤10 years)	30 (18.2%)	25 (17.1%)	ns
Secondary level (11–13 years)	92 (55.8%)	85 (58.2%)	ns
Tertiary level (>13 years)	43 (26%)	36 (24.7%)	ns
N. of DMTs switch pre-progression	0.3 ± 0.6	0.4 ± 0.7	ns
Patients on interferon beta 1b at the time of SPMS conversion *n* (%)	72 (43.6%)	76 (52%)	ns
Patients on other DMTs *n* (%)	93 (56.4%)	70 (48%)	ns
Follow-up duration (months)	79.3 ± 18.7	85.3 ± 23.5	ns
EDSS at disease progression (median; min-max)	4.5 (4.0–5.5)	5.0 (4.5–5.5)	ns

* Data are on mean ± SD, unless otherwise specified; DMTs: disease modifying therapy; EDSS: Expanded Disability Status Scale; SPMS, secondary progressive multiple sclerosis.

## Data Availability

Anonymized data will be shared upon request from any qualified investigator for the sole purpose of replicating procedures and results presented in the report, provided that the data transfer is in agreement with EU legislation on the general data protection regulation. All researchers could access all the data.
